# Surgical management of the colorectum in FAP: tailored approaches for optimal outcomes

**DOI:** 10.1007/s10689-025-00492-6

**Published:** 2025-09-05

**Authors:** A. Sinha, J. G. Karstensen, D. Liska

**Affiliations:** 1 St Mark’s Centre for Familial Intestinal Cancers, St Mark’s, The National Bowel Hospital, London, UK; 2 Danish Polyposis Register, Gastro Unit, Copenhagen University Hospital - Amager and Hvidovre, Hvidovre, Denmark; 3 Department of Clinical Medicine, University of Copenhagen, Copenhagen, Denmark; 4 Department of Colorectal Surgery, Digestive Disease Institute, Cleveland Clinic, 9500 Euclid Avenue / A30, Cleveland, OH 44195 USA; 5 Sanford R. Weiss, M.D. Center for Hereditary Colorectal Neoplasia, Cleveland Clinic, Cleveland, OH USA

**Keywords:** Familial adenomatous polyposis, Colorectal neoplasm/prevention and control, Proctocolectomy, Restorative, Ileorectal anastomosis, Minimally invasive surgical procedures

## Abstract

Familial adenomatous polyposis (FAP) is an inherited condition that predisposes individuals to colorectal cancer without preventive treatment. Surgical management typically involves restorative proctocolectomy with an ileal pouch anal anastomosis or colectomy with ileorectal anastomosis. Complete removal of the large intestine and rectum with a permanent stoma may also be required in selected cases. This narrative review highlights decision-making in FAP regarding the timing, extent, and modality of large bowel surgery. Key considerations include the extent of polyps, cancer risk in the remaining rectum, and associated extra-colonic manifestations like desmoid disease. The timing of surgery and the extent of bowel removal are critical factors requiring a personalized approach that considers patient preferences and clinical factors. Regardless of the chosen strategy, continued surveillance is essential to monitor disease progression.

## Introduction

Familial adenomatous polyposis (FAP) is a dominantly inherited condition caused by pathogenic variants in the *APC* tumour suppressor gene which inevitably progresses to cancer if left untreated [1, 2]. More than 825 germline mutations have been described in the *APC* mutation database and mutation analysis is successful in approximately 70–95% of patients with FAP [3–5]. The surgical management of familial adenomatous polyposis (FAP) is a critical component in reducing the risk for colorectal cancer in affected individuals. This narrative review regarding the colorectal surgical management of FAP is not intended to serve as an exhaustive summary of the literature or a restatement of existing guidelines. Rather, it offers a pragmatic, experience-based approach as practiced by the authors and their institutions, grounded in decades of collective expertise within the context of some of the largest hereditary colorectal cancer registries.

Currently, most patients with FAP are diagnosed before the development of symptoms by screening, either genetic or endoscopic, predicated by a family history of FAP [6]. For patients with a known diagnosis of FAP, colorectal surveillance with colonoscopies is usually recommended to start at age 10–12 years old [7, 8].Since the risk for colorectal cancer in FAP approaches 100%, risk-reducing large bowel surgery remains the cornerstone of treatment and prophylaxis against colorectal cancer in patients with FAP. Restorative proctocolectomy (RPC) with or without mucosectomy, and total colectomy with ileorectal anastomosis (TC-IRA) represent the traditional restorative surgical options for these patients. Panproctocolectomy with an end ileostomy, remains an alternative when mesenteric desmoid disease precludes RPC or a patient has poor sphincter function. In cases of malignant disease identified at the time of diagnosis, oncological principles are strictly adhered to.

## Genotype-phenotype correlations

Patients with an *APC* germline mutation around codon 1309 have an exceptionally severe phenotype, with high polyp burden, early development of cancer and a high risk of progressive disease in the retained rectum if an IRA is performed [9]. Germline mutations 5′ of codon 160 and 3′ of codon 1450 are associated with an attenuated phenotype, with later onset of scantier adenomas [10]. Patients with 3′ mutations have also been shown to have an increased risk for desmoid formation [11]. However, it is important to note that genotype-phenotype correlations are far from universal and even family members with the same *APC* variant can present with different phenotypes. It is therefore essential to carefully assess each patient’s colonic polyposis phenotype before making treatment recommendations. It has been suggested that in patients with an attenuated phenotype polyposis (usually defined as less than 100 colorectal polyps by age), penetrance for colorectal cancer is less than 100%, and that some patients with a light polyp burden and an attenuated course of disease may be managed endoscopically, without the need for prophylactic surgery. Often endoscopic surveillance can safely be initiated in the late teens [12]. Increasingly, appreciation of a patient’s genotype, phenotype along with current or future desmoid risk is used to develop a bespoke surgical strategy in conjunction with the patient’s lifestyle, medical co-morbidities, anal sphincter function and continence, and access to high quality on-going endoscopic surveillance.

### Decision making for risk-reducing colectomy

Surgery has significant implications on the patient’s risk for cancer and quality of life. Therefore, it is of utmost importance that a provider with experience in the management of FAP discusses all salient factors with the patient to facilitate shared decision-making. The two most important initial decisions to make with regards to risk-reducing large bowel surgery revolve around the timing of surgery, meaning at what age surgery should be performed, and the extent of surgery, meaning how much of the large bowel should be removed at the time of surgery.

#### Timing of risk-reducing colectomy

When discussing the timing of large bowel surgery for patients with FAP, it is important to frame the discussion around the primary goals of surgery which are to prevent cancer, and more specifically death from cancer, while at the same time maximizing the quality of life for the patient. Prophylactic surgery should be performed with as little complications as possible and regular audit and review of clinical outcomes in useful.

It is relevant to note that colorectal cancer even in patients with FAP is a rare occurrence before the age of 20 years. In a survey of 26 different registries affiliated with the Leeds Castle Polyposis Group conducted by Church et al., there were only 14 patients identified as having invasive colorectal cancer younger than 20 years, with only one being diagnosed before the age of 15 years [13]. Since colorectal surgery has the potential to cause major disruption in a teenager’s life, especially if surgery entails a temporary ostomy, one will ideally postpone surgery to a time where the impact on normal adolescent and young adult physiologic and psychosocial development is minimal, provided there is no significant increase in the risk of cancer.

The risk for colon and rectal cancer can be predicted by the genotype and more importantly the colorectal polyposis phenotype of the patient. It is therefore critically important for clinicians performing colonoscopies in FAP to meticulously document endoscopic findings including the size, number, and location of polyps, including an explicit description of the polyposis burden in the rectum, and the proximity of polyps to the dentate line. While finding a cancer is clearly an absolute indication for surgery, other relative indications to proceed with surgery within a short time following colonoscopy include the detection of advanced adenomas (high grade dysplasia or adenomas > 10 mm), symptomatic polyposis, profuse polyposis or carpeting of polyps that preclude safe endoscopic surveillance, or a patient with difficulty adhering to prescribed endoscopic surveillance intervals. Factors in favor of deferring surgery include a low polyp burden with only small polyps without any advanced dysplasia, good adherence with prescribed endoscopic surveillance intervals, physiologic or psychologic immaturity that would make recovery from surgery more difficult (especially if a total proctocolectomy is required), and social or academic circumstances that would be significantly affected by surgery and recovery.

The risk of desmoid disease is another factor that is becoming increasingly recognized as a reason to defer risk-reducing surgery when possible [14]. This is because the majority of intra-abdominal desmoids arise after abdominal surgery, and in some patients these tumors cause great suffering and even death. Patients at risk can be recognized by genotype (mutations 3′ of codon 1444) and phenotype (strong family or personal history of desmoid disease, or extra-abdominal desmoids diagnosed before abdominal surgery). Many patients at high risk for desmoids have a milder polyposis phenotype, providing latitude to defer surgery. Whenever surgery is deferred for one of these reasons, surveillance colonoscopy should continue at yearly intervals pending findings.

There are several recent studies from hereditary registries reporting outcomes regarding the timing of surgery in FAP [15–17]. In a cohort from the Cleveland Clinic Jagelman Registries including 211 patients with FAP who had their first colonoscopy under the age of 30 years old between 2000 and 2017, 45% had undergone surgery at a mean age of 20.1 years old [16]. While 7 patients were diagnosed with cancer on their initial colonoscopy, among patients under endoscopic surveillance, there was only 1 patient diagnosed with cancer at the age of 29 years. On analysis of factors associated with the recommendation to proceed with surgery, genotype (e.g. pathogenic variant within the mutation cluster region between codons 1250 and 1450), greater that 100 polyps, and any polyp with high-grade dysplasia were found to be associated with a higher likelihood of undergoing surgery within 2 years. A family history of desmoid disease and the use of chemoprevention were found to be associated with a lower likelihood of undergoing surgery within 2 years.

In a study from the St. Mark’s Polyposis Registry including 84 pediatric FAP patients having their first colonoscopy under the age of 18 years, 45 patients had undergone surgery at a median age of 17 years, with the most common indications for surgery being cited as social convenience (51%), patient/family preference (33%), and increase in polyp burden or size (16%) [15]. Importantly, the authors concluded that most patients underwent surgery as a planned procedure at a time that was least disruptive to the child’s social and educational development. These, and similar studies demonstrate that at expert centers selecting appropriate patients for deferral of surgery is a safe practice. Therefore, when contemplating optimal timing of surgery in young people with FAP, a thoughtful approach balancing the risk for cancer and the overall impact of surgery is critical to achieve the best possible outcomes [18].

#### Extent of risk-reducing colectomy

Once the decision to proceed with risk-reducing large bowel surgery is made, several factors must be carefully considered when choosing the appropriate extent of surgery. As discussed before, the goal of surgery is to prevent or treat colorectal cancer while preserving gastrointestinal function and maintaining the patient’s quality of life. Since prophylactic colectomy does not cure FAP, nor fully eliminate the risk of gastrointestinal cancer, the surgical approach ideally strikes a balance between minimizing cancer risk while ensuring an optimal quality of life. This is particularly important for patients who are usually asymptomatic, young, and part of a family where the outcome of one patient’s surgery may influence other relatives’ adherence with treatment recommendations.

The two main colorectal surgical options for FAP are total abdominal colectomy with ileorectal or ileosigmoid anastomosis (IRA) and restorative proctocolectomy with an ileal pouch-anal anastomosis (RPC). In rare cases where restoring intestinal continuity is not desired, a panproctocolectomy with end ileostomy (PPC) may be considered. These days, most units would offer minimally invasive surgery and deploy modern best-practices such as enhanced recovery after surgery. Some centers recommend RPC for all patients with FAP due to its highly effective reduction in colorectal cancer risk and overall positive outcomes. However, even at expert centers RPC can be associated with significant postoperative morbidity, often requires a temporary ileostomy and can be associated with a reduction in fecundity in females, damage to pelvic nerves controlling sexual function, and an increase in the risk for intraabdominal desmoid disease. When comparing gastrointestinal functional outcomes, a meta-analysis of observational studies of IRA versus RPC for FAP including 1,002 patients found that bowel frequency, night defecation, and incontinence were significantly more common in the RPC group [19].

## *Total abdominal colectomy and ileorectal or ileosigmoid anastomosis*

Given these factors, it is logical to consider colectomy and IRA when possible, as preserving the rectum avoids the increased risk for serious postoperative complications, issues related to pelvic dissection like fertility and sexual function, does not require a temporary stoma, and offers more predictable bowel function. Selecting patients for rectal preservation is based on several factors but primarily guided by the likelihood of requiring a proctectomy later in life and the risk for rectal cancer. Typically, IRA is recommended for individuals with a mild polyposis phenotype—fewer than 20 rectal polyps and fewer than 1000 colonic polyps—which goes along with a genotype where the *APC* pathogenic variant is outside the mutation cluster region. However, other considerations, such as the patient’s plans to have children, obesity, and the ability to adhere to regular screening, must also influence the decision. Recent data from the Cleveland Clinic and the European FAP Consortium have shown that the risk for desmoid tumors and especially intraabdominal desmoid disease is significantly higher following RPC when compared to IRA [14, 20]. Selection of the extent of surgery therefore requires a personalized and nuanced approach, considering a multitude of factors. Not all patients with more than 20 rectal polyps are best served by RPC and IRA may not be the best option for every patient with mild polyposis.

The main drawback of rectal preservation in FAP is the risk of progression of polyposis and development of cancer in the rectum. There are multiple studies that have assessed the risk of requiring proctectomy or rectal cancer following IRA for FAP. However, it’s important to note that many of the publications indicating a higher need for proctectomy following IRA include patients from the pre-pouch era, when rectal preservation was limited by the lack of restorative options after proctectomy leading to the selection of patients with a relatively high rectal polyp burden for IRA [21]. A study from the St. Mark’s Polyposis Registry following 427 FAP patients who underwent IRA (with around 50% of patients from the pre-pouch era) found that proctectomy was required in 29% and that rectal cancer developed in 11% over a median follow-up of 15 (range 7–25) years. By the age of 60 years half of the patients retained their rectum [22]. Importantly, almost 80% of patients with an IRA who required a proctectomy, were still able to undergo restorative surgery. A more recent study from the Cleveland Clinic Jagelman Registries, focusing on 197 patients selected for IRA in the pouch era (1993–2020) found that the risk for proctectomy and rectal cancer were only 8% and 3% respectively with a median follow-up after IRA of 13 years (interquartile range, 6–17) [23]. A recent European multicenter study including 865 FAP patients found that the 20-year risk of developing cancer after colectomy with IRA was 2.5% vs. 0.9% for RPC [24]. Factors associated with rectal failure from these studies include *APC* pathogenic variants in the mutation cluster region, greater than 20 rectal polyps or greater than 500 colon polyps at the time of surgery, and rectal polyps with high-grade dysplasia. In patients without any of these risk factors, IRA is an excellent risk-reducing surgery affording good quality of life while also minimizing the risk for cancer, provided the patient remains adherent with close endoscopic surveillance.

Anastomotic leakage rates of 11% have been reported following IRA. Ileo-distal sigmoid anastomosis (IDSA) is a recent modification. In a study from St Mark’s Hospital Polyposis Registry, a total of 191 patients with FAP underwent laparoscopic colectomy between 2006 and 2017, of whom 139 (72.8%) underwent an IRA and 52 (27.2%) an IDSA [25]. The median age at surgery in the IRA and IDSA groups was 20 years (IQR 17–45) and 27 years (IQR 19–50), respectively. Grade II complications were comparable between the two groups. There were no anastomotic leakages in the IDSA group compared with 15 (10.8%) in the IRA group (*P* = 0.0125) and no reoperation in the IDSA group compared with 17 (12.2%) in the IRA group (*P* = 0.008). The frequency of polypectomies per flexible sigmoidoscopy was comparable between the two groups indicative of appropriate patient selection for IDSA. These improved outcomes after an IDSA are thought to be a function of the preservation the inferior mesenteric artery during the colectomy and adoption of a side-to-side stapled IDSA, alongside centralizing surgery to high-volume surgeons.

## *Restorative proctocolectomy*

When rectal preservation is not a good option, a further decision regarding the extent of surgery when performing a RPC needs to be considered with respect to the two main anastomotic options, a mucosectomy with hand-sewn anastomosis or a stapled anastomosis. A mucosectomy ostensibly removes the entire colorectal mucosa at risk for cancer, while a stapled anastomosis leaves behind a short rectal cuff of residual rectal mucosa at the anal transitional zone (ATZ). Deciding between these two options again revolves around balancing quality of life considerations with the risk for rectal cuff/ATZ polyp progression and cancer. Studies have shown that a stapled anastomosis is associated with improved bowel function (significantly less seepage and incontinence) and decreased anastomotic complications when compared to a mucosectomy with handsewn anastomosis [26]. A recently published systematic review including 8872 patients undergoing IPAA for FAP and inflammatory bowel disease found that a stapled anastomosis was associated with lower rate of anastomotic stricture, small bowel obstruction, pouch failure, decreased seepage, pad use, and nocturnal incontinence [27]. While the incidence of ATZ adenomas is significantly higher in patients with stapled anastomoses, a mucosectomy does not eliminate the risk for ATZ adenomas. Furthermore, the risk for ATZ cancers has not been shown to been shown to be significantly different between patients with stapled or handsewn anastomoses, provided that patients undergo close surveillance and endoscopic or transanal management of ATZ polyps [28]. To optimize functional outcomes following RPC, it is therefore our preferred practice to perform a stapled anastomosis if the very distal rectum and ATZ are relatively spared of polyps at the time of ileal-pouch anal anastomosis construction. To minimize the risk of complex neoplasia developing at the ATZ, a technically sound IPAA construction with a very short rectal cuff, experienced endoscopic surveillance and management of incident ATZ polyps, alongside excellent patient adherence to surveillance are all critically important.

## Minimally invasive surgery

Multiple cohort studies and multicenter series have shown that laparoscopic surgery in FAP is feasible, with low conversion rates, acceptable morbidity, and no increase in long-term cancer risk compared to open surgery. Laparoscopic surgery is particularly advantageous in young patients typical of FAP, especially considering the increased risk for needing further abdominal surgery in the future. For individuals requiring RPC, the introduction of robotic surgical platforms has enabled enhanced vision and dexterity in the pelvis, along with the possibility of performing an intracorporeal double purse-string anastomosis (robotic intracorporeal single-stapled anastomosis- RiSSA) [29, 30]. Early experience with this technique has led to a further reduction in anastomotic leak rate, improved functional outcomes, decreased use of fecal diversion and no significant alteration in polyp burden within the cuff of the pouch [31]. Because a restorative RPC requires multi‑quadrant access, a hybrid robotic approach can be employed to combine the advantages of robotic surgery in the pelvis with a laparoscopic abdominal colectomy, thereby avoiding the need for multiple robotic redockings [32].

### Postoperative surveillance after colectomy with pouch or ileorectal anastomosis

Postoperative surveillance in FAP patients remains critical due to the persistent risk of adenoma and cancer development in the remaining rectal or ileal mucosa. The choice between IRA and IPAA influences the surveillance protocol and risk profile [24]. Patients undergoing IRA retain their rectum, necessitating regular endoscopic surveillance to monitor for adenomas or malignant transformation. Guidelines typically recommend annual colonoscopy with interventions such as polypectomy or ablative techniques for adenomas larger than 5 mm (Table 1) [7, 8, 33–36]. Although less common, cancers in the pouch body or anastomosis have been reported, emphasizing the need for routine surveillance [37]. Endoscopic surveillance for IPAA patients typically involves annual pouchoscopy, focusing on areas prone to adenomas, such as the ileal mucosa and anastomotic sites. Retroflexed views are recommended to ensure thorough examination anal of the anal cuff. International guidelines generally recommend annual or biannual endoscopy, with tailored intervals based on individual risk factors and prior findings (Table 1) [7, 8, 34–36]. To evaluate efficacy and safety of postoperative practice, a European prospective multicenter study is currently investigating a more individualized surveillance protocol [38].

Ultimately, long-term surveillance is essential to prevent morbidity and mortality from secondary malignancies in FAP patients following colectomy, underscoring the need for adherence to rigorous protocols. Although beyond the scope of this review, it is important to highlight that, in addition to lower gastrointestinal surveillance, patients with FAP also require structured surveillance of the stomach and duodenum, as well as monitoring for thyroid disease and desmoid tumors.

In conclusion, surgical management of the colorectum in FAP requires a personalized approach balancing cancer risk, surgical morbidity, and patient preferences. The timing and choice of risk-reducing procedure depend on genotype and polyposis phenotype, cancer risk, extra-colonic disease and quality of life implications. Regardless of the extent of surgery and approach, lifelong surveillance at specialist centers—equipped with the expertise to manage ongoing polyp burden and extraintestinal manifestations—remains critical to detecting and managing disease progression and optimizing outcomes.

**Table 1:** Postoperative surveillance of patients with familial adenomatous polyposis

	ASGE	ESGE	ACG	EHTG-ESCP	BSG-ACPGBI-UKCGG
Interval / years					
Remnant rectum	0.5-1	1–2	1	1–2	1–3
Ileal pouch	1–2	1–2	1	1	1–3

ASGE, American Society of Gastrointestinal Endoscopy; ESGE, European Society of Gastrointestinal Endoscopy; ACG, American College of Gastroenterology; EHTG, European Hereditary Tumour Group; ESCP, European Society of Coloproctology; BSG, British Society of Gastroenterology; ACPGBI, Association of Coloproctology of Great Britain and Ireland; UKCGG, United Kingdom Cancer Genetics Group.

## Data Availability

No datasets were generated or analysed during the current study.
